# “Switch-Off” of Respiratory Sinus Arrhythmia May Be Associated With the Activation of an Oscillatory Source (Pacemaker) in the Brain Stem

**DOI:** 10.3389/fphys.2019.00939

**Published:** 2019-07-30

**Authors:** Gert Pfurtscheller, Beate Rassler, Andreas R. Schwerdtfeger, Wolfgang Klimesch, Alexandre Andrade, Gerhard Schwarz, Julian F. Thayer

**Affiliations:** ^1^Institute of Neural Engineering, Graz University of Technology, Graz, Austria; ^2^BioTechMed-Graz, Graz, Austria; ^3^Carl-Ludwig-Institute of Physiology, University of Leipzig, Leipzig, Germany; ^4^Institute of Psychology, University of Graz, Graz, Austria; ^5^Centre of Cognitive Neuroscience, University of Salzburg, Salzburg, Austria; ^6^Instituto de Biofísica e Engenharia Biomédica, Faculdade de Ciências da Universidade de Lisboa, Lisbon, Portugal; ^7^Division of Special Anaesthesiology, Pain and Intensive Care Medicine, Department of Anaesthesiology and Intensive Care Medicine, Medical University of Graz, Graz, Austria; ^8^Department of Psychological Science, The University of California, Irvine, CA, United States

**Keywords:** respiratory sinus arrhythmia, heart rate variability, 0.1-Hz oscillations, blood-oxygen level-dependent activity, brain stem, central pacemaker

## Abstract

Recently, we reported on the unusual “switch-off” of respiratory sinus arrhythmia (RSA) by analyzing heart rate (HR) beat-to-beat interval (RRI) signals and respiration in five subjects during a potentially anxiety-provoking first-time functional magnetic resonance imaging (fMRI) scanning with slow spontaneous breathing waves (Rassler et al., 2018). This deviation from a fundamental physiological phenomenon is of interest and merits further research. Therefore, in this study, the interplay between blood-oxygen level-dependent (BOLD) activity in the cerebellum/brain stem, RRI, and respiration was probed. Both the cardiovascular and the respiratory centers are located in the medulla oblongata and pons, indicating that dominant slow rhythmic activity is present in the brain stem. The recording of BOLD signals provides a way to investigate associated neural activity fluctuation in the brain stem. We found slow spontaneous breathing waves associated with two types of slow BOLD oscillations with dominant frequencies at 0.10 and 0.15 Hz in the brain stem. Both BOLD oscillations were recorded simultaneously. One is hypothesized as vessel motion-based phenomenon (BOLDv) associated with the start of expiration; the other one as pattern associated with neural activity (BOLDn) acting as a driving force for spontaneous inspiration and RRI increase (unusual cessation of RSA) about 2–3 s after BOLDv. This time delay of 2–3 s corresponds to the neurovascular coupling time.

## Introduction

Respiratory sinus arrhythmia (RSA) reflects heart rate (HR) acceleration during inspiration and HR deceleration during expiration. It is a fundamental principle (Yasuma and Hayano, 2004) and the core phenomenon of paced resonance breathing at 6/min associated with amplified low frequency heart rate variability (HRV), heightened emotional well-being (Mather and Thayer, 2018), and improved processing of negative emotions (Zaccaro et al., 2018). Interestingly, there are exceptions from RSA with HR beat-to-beat interval (RRI) increases during inspiration and with RRI phase leading relative to respiratory rhythm (Rassler et al., 2018). This unusual paradoxical RSA was found in a minority of healthy participants during functional magnetic resonance imaging (fMRI) scanning, an uncomfortable, sometimes claustrophobic situation usually associated with increased state anxiety (Munn et al., 2015; Pfurtscheller et al., 2018).

Slow spontaneous breathing waves with a rate between 6 and 9 breaths/min are also accompanied by elevated HRV and could thus facilitate the processing of unpleasant emotions like anxiety and stress (Thayer and Lane, 2009). Due to the leading role of RRI over breathing oscillations during the unusual cessation of RSA (Rassler et al., 2018), an autonomous neural oscillator (central pacemaker; Julien, 2006) in the brain seems likely, which acts as a source for slow RRI oscillations. It is noteworthy that Perlitz et al. (2004) reported on a new type of cardiovascular rhythm in the 0.15-Hz band in man and dog with a common origin in the brain stem. The most interesting feature of this broad band “0.15-Hz rhythm”(including also frequency components close to 0.1 Hz) is that periods of spindle waves are phase-coupled with respiration at a ratio of 1:1. Our chief research interest was to identify this pacemaker, which is suggested to be located in the brain stem (Lambertz and Langhorst, 1998; Perlitz et al., 2004), by analyzing BOLD signals.

The BOLD signal does not directly quantify neural activity itself but is sensitive to changes in the cerebral metabolic rate, cerebral blood flow, and cerebral blood volume (Obrig et al., 2000; Buxton et al., 2004) and to several types of motion (chest and blood vessel movements; Birn et al., 2006). Therefore, BOLD signals can be composed of neural and non-neural (e.g., vessel motion) components. It is expected that two BOLD signals of different origin could be identified in the brain stem with its large blood vessel (basilar artery) and the postulated neural 0.15-Hz source (Perlitz et al., 2004). We assume that one of them results from blood vessel motion (BOLDv) and indicates the start of a slow respiratory action as it occurs almost simultaneously with neural activation of respiratory neurons. The other one is thought to be associated with neural activation (BOLDn) delayed by the neurovascular coupling time of 2–3 s (Mateo et al., 2017). We speculate that both BOLD signals point to a central pacemaker in the brain stem.

## Materials and Methods

### Subjects and Experimental Paradigm

The group of subjects studied was the same (age 23.8 ± 3.3 years) as analyzed recently by Rassler et al. (2018). Recording and preprocessing of ECG and respiration were described in that paper. All participants gave informed written consent to the protocol of the study, which had been approved by the local Ethics Committee at the University of Graz.

### Functional Magnetic Resonance Imaging and Blood-Oxygen Level-Dependent Signals

Functional images were acquired with a 3 T scanner (Magnetom Skyra) using a multiband GE-EPI sequence (Moeller et al., 2010) with a simultaneous six-band acquisition with TE/TR = 34/871 ms, 52° flip angle, 2 mm × 2 mm × 2 mm voxel size, 66 contiguous axial slices (11 × 6), acquisition matrix of 90 × 104, and a FOV of 180 mm × 208 mm. This scanning rate of 871 ms (1.15 Hz sampling frequency) allows studying slow oscillations in the range 0.1–0.15 Hz. For further details about preprocessing, see Pfurtscheller et al. (2018). The automated anatomical labeling (AAL) atlas (Tzourio-Mazoyer et al., 2002) was used to extract time courses of BOLD signals in 116 regions of interest (ROIs). This atlas shows no ROIs in the brain stem, but only in close proximity of cerebellum and vermis. Due to the proximity of these regions and due to the contiguity of a major artery, it is reasonable to assume that the signal collected from AAL ROIs labeled “cerebellum” partially reflects brain stem activity.

### Selection of Regions of Interest for Blood-Oxygen Level-Dependent Signals From Cerebellum/Brain Stem

**Figure 1:**
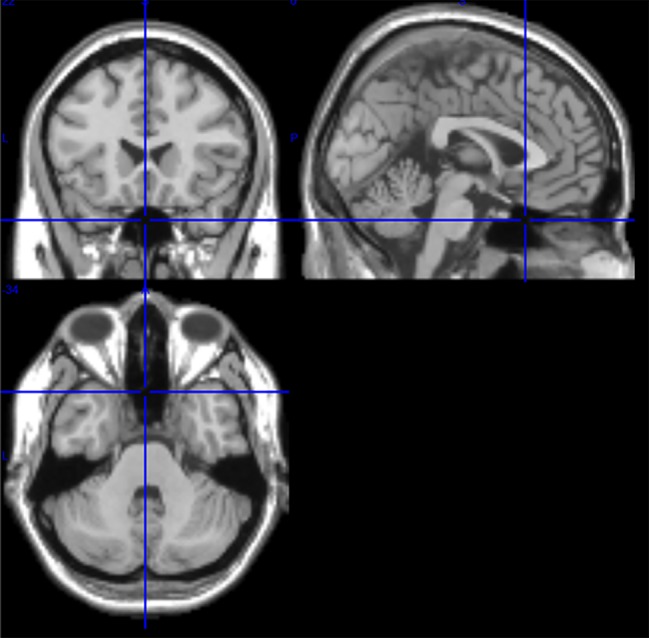
T1 images from medial, sagittal, and axial slices from one subject (Talairach space 0, 20, −34). In the sagittal slice (upper right image), the pons is clearly visible, and in the axial slice (bottom) the proximity of cerebellum and brain stem is documented.

Among the 116 ROIs of the AAL atlas, ROI 91 to ROI 108 correspond to axial slices from the cerebellum including the brain stem (Tzourio-Mazoyer et al., 2002). T1-images from the axial slice (Talairach space *z* = −34) document the proximity of cerebellum and brain stem (Figure 1). Due to their small number of voxels (<200), ROIs 107 and 108 were excluded from further analysis. ROIs from the cerebellum/brain stem were visually inspected in order to search for appropriate ROIs, i.e., ROIs with a good signal-to-noise ratio and dominant slow BOLD waves. The following ROIs were identified: 96, 98, 100, 103, and 105. The numbers indicate the ROI labels according to the AAL atlas. Odd and even numbers denote left and right hemispheres, respectively.

### Calculation of Averaged Blood-Oxygen Level-Dependent, Beat-to-Beat Interval, and Breathing Waves

**Figure 2:**
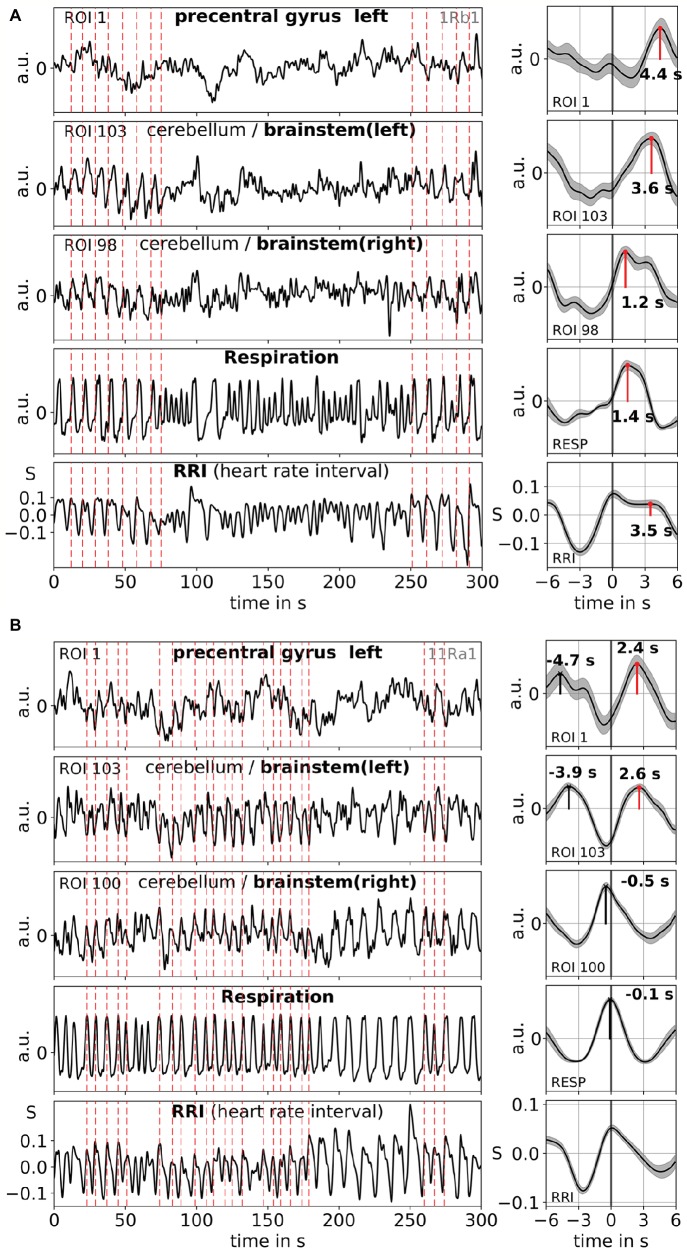
Examples of ongoing BOLD, RRI, and respiration signals and corresponding averaged waves (±SE) for two subjects, one with dominant 0.1-Hz [1Rb1 **(A)**] and one with dominant 0.15-Hz oscillations [11Ra1 **(B)**]. The vertical dashed lines in the panels on the left-hand side indicate maxima (peaks) of RRI oscillations used as trigger for averaging. Peaks of the averaged waves are indicated in the panels on the right-hand side.

Averaging allows enhancing the signal-to-noise ratio but requires the use of a trigger. Because no such triggers are available in resting state data, the rhythmically occurring maxima (peaks) of the RRI signal were used (Pfurtscheller et al., 2017): First, the most prominent peaks of the RRI signals, spaced at least several seconds apart, were identified. In the case of 0.1-Hz oscillations, these intervals between the peaks were around 10 s, and in the case of 0.15-Hz oscillations, they were around 7 s (an example is depicted in Figure 2 by the almost equidistant dashed vertical lines). The marked RRI peaks were used as triggers for averaging (epochs with 6 s before and 6 s after the trigger) across the BOLD, RRI, and respiration signals.

## Results

Slow BOLD oscillations in the cerebellum/brain stem are characterized by their favorable signal quality but even more importantly, by their coincidence with oscillations in RRI and respiration. As reported in Rassler et al. (2018), 0.1-Hz oscillations prevailed during ~37% of the recording time, while 0.15-Hz oscillations prevailed in ~45%. Two characteristic examples for 0.1-Hz (subject 1Rb1) and 0.15-Hz (subject 11Ra1) oscillations are displayed in Figure 2. In both cases, RRI oscillations of large magnitude coincide with respiration in form of an RRI increase during inspiration and are also clearly reflected in the BOLD signals in brain stem. Note, the varying dynamics of spontaneous oscillations and single waves, respectively, in the resting state give strong evidence that not only one but a variety of cardiovascular rhythms exist with frequency components close to 0.1 and 0.15 Hz. Some of these rhythms are amplified during anxiety processing, some not, and some are synchronized in prefrontal cortex and brain stem and some not.

**Figure 3:**
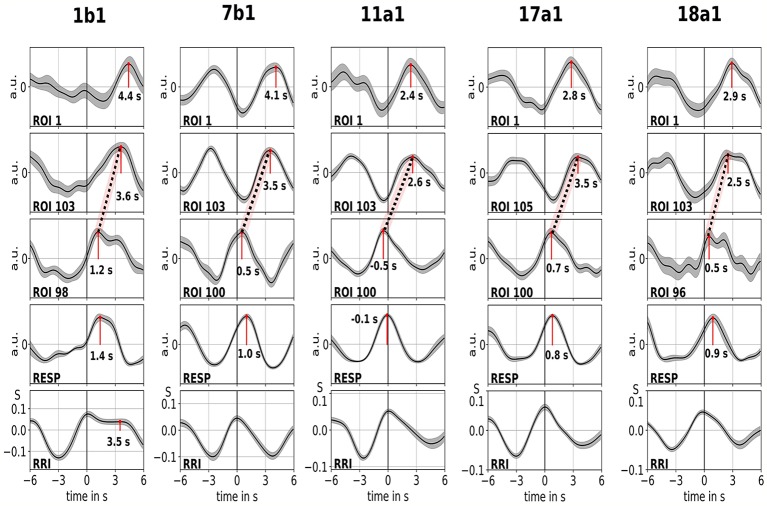
Averaged waves (±SE) of BOLD signals from left precentral gyrus (ROI 1), left brain stem (BOLDn, ROIs 103, 105), right brain stem (BOLDv, 96, 98, 100), respiration, and RR intervals (from top to bottom) from all five subjects. The peak latencies (difference from RRI peak) from important peaks are marked. The time shift of 2–3 s between the two BOLD signals from the cerebellum/brain stem is indicated by a dotted line.

Examples of spontaneous signals and averaged waves of two characteristic subjects are shown in Figure 2. The averaged waves with marked peak latencies (distance from RRI peak) of all subjects are displayed in Figure 3. Of note, the relatively small variance of the averaged BOLD waves in the brain stem underlines the reliability of the peak measurements.

The BOLD wave in the right hemisphere preceding the breathing wave by 0.3 ± 0.2 s was interpreted as BOLDv, and the BOLD wave in the left hemisphere lagging the breathing wave by 2.3 ± 0.5 s was taken as BOLDn. The peak difference between BOLDv and BOLDn waves was 2.6 ± 0.4 s.

The averaged waves in Figure 3 highlight the clear phase-shift between the two BOLD signals in the brain stem (BOLDn, BOLDv) in all five subjects. Peak differences are indicated by dotted lines. In addition to the two BOLD signals from the brain stem, the BOLD signal from left precentral gyrus (ROI 1) was analyzed as control. Although the averaged BOLD waves in the prefrontal cortex (PFC) display an elevated variance (SE), they are in surprisingly perfect match with the BOLDn waves in the cerebellum/brain stem. This confirms the strong interaction between PFC and brain stem in the slow frequency range around 0.1 Hz.

## Discussion

### Coincidence of Breathing Waves and Respiratory Blood-Oxygen Level-Dependent Artifact

Breathing is accompanied not only by chest motion but also by motion of cerebral blood vessels (Birn et al., 2006). A stable time shift of 0.3 ± 0.2 s was observed between the positive peak of the BOLDv signal in the right cerebellum/brain stem and the maximum of each breathing wave (start of expiration). Respiration was spontaneous, and the respiration peak lagged the BOLDv peak, possibly indicating a brisk vasomotion of the basilar artery, a large blood vessel close to the rostral side of the pons. This vasomotion might be considered a respiratory artifact induced by a respiratory modulation of sympathoexcitatory neurons in the rostroventrolateral reticular nucleus. A marked influence of the central respiratory pattern generator upon sympathetic nerve activity has been observed in many species including the human (Haselton and Guyenet, 1989; Häbler and Jänig, 1995; Eckberg, 2003; Mandel and Schreihofer, 2006). Decreasing activity of these neurons in the rostroventrolateral medulla (i.e., sympathetic depression) during inspiration and an abrupt re-increase of activity with cessation of phrenic bursts is one of the predominant patterns of respiratory-sympathetic coupling (Haselton and Guyenet, 1989). These respiration-modulated sympathetic oscillations cause maximal vasodilatation at the end of inspiration (i.e., maximum of the BOLD signal immediately preceding the start of expiration) and vasoconstriction during expiration (minimum of BOLD signal just preceding the onset of inspiration).

### Rhythmic Neural Activation Associated With Neural Blood-Oxygen Level-Dependent Oscillation

BOLD oscillations (BOLDn) lagged the periodic respiratory artifact (BOLDv) by 2.6 ± 0.4 s. Both BOLD oscillations were recorded simultaneously in axial slices with BOLDn being associated with neural activity as a driving force for spontaneous breathing and BOLDv reflecting a vessel motion associated with the start of expiration. This time delay of 2–3 s corresponds to the neurovascular coupling time (Mateo et al., 2017).

Various external influences on emotion elicited by the uncomfortable supine position with the subject’s head in a limited noisy space may activate primary sensory areas first and be followed by cortical projection to brain stem respiratory neurons. Moreover, internal, behavioral influences arising from higher centers modify metabolic breathing patterns. The final respiratory output involves a complex interaction between cortical structures, brain stem, and limbic system and is characteristic for emotional breathing (Homma and Masaoka, 2008; Kato et al., 2018). Therefore, it is quite plausible that oscillations in the left precentral gyrus (ROI 1) were synchronized with the slow, neurally induced BOLD oscillations in the cerebellum/brain stem.

### Neural Activity Act as “Driving Force” for Slow Heart Rate Oscillations

The varying dynamics of the coupling patterns observed in the resting state point toward a variety of cardiovascular rhythms in the frequency band between 0.1 and 0.15 Hz. This underlines the importance, flexibility, and complexity of brain-heart interaction and merits further intensive research.

In all five participants, the coupling pattern between respiration and HR was quite contrary to typical RSA (Rassler et al., 2018), namely the RRI increased during inspiration and decreased during expiration. During anxiety-provoking situations, breathing tends to be faster and vagal activity diminished, leading to a higher HR (shorter RRI). However, when breathing is unconsciously/autonomically slowed down in an anxiety-provoking situation, vagal activity could increase, resulting in a lower HR (larger RRI). It seems that not only cortical activation induces an almost simultaneous RRI increase (Barry, 1983; Damen and Brunia, 1987; Pfurtscheller and Lopes da Silva, 1999; Pfurtscheller et al., 2013), but our study provides first evidence that also a central pacemaker in the cerebellum/brain stem can act as driving force for intrinsic RRI oscillations and spontaneous slow breathing waves. This finding strongly supports the work of Perlitz et al. (2004) on the “0.15-Hz rhythm.”

### Limitations and Future Prospects

The selection of ROIs with good quality BOLD signals in cerebellum/brain stem was made by visual inspection. This included differentiation between BOLD oscillations of neural origin and motion artifacts. The latter denotes BOLD signals time-locked with respiration. For further studies, it is recommended to calculate phase-locking values (PLV; Pfurtscheller et al., 2017) between RRI and individual BOLD signals in the cerebellum/brain stem. Additionally, the synchronous evaluation of ventilation metrics, such as tidal volume/respiratory volume per time, end-tidal pCO_2_, and type of breathing (nose vs. mouth), as well as the use of various denoising methods in clinical and animal experimental settings, could give deeper insights into the complex interactions of the involved regulatory systems.

The “switch-off” of respiratory sinus arrhythmia is an exception from a fundamental physiological phenomenon that may occur, e.g., in anxiety-provoking situations. Among 23 healthy participants of an fMRI study, only five subjects presented this paradoxical coupling pattern.

A prerequisite for BOLD (Bn and Bv) analysis in brain stem is a coincidence of slow breathing and RRI oscillations; however, such a 1:1 coupling can only be found in a minority of participants. Hence, this kind of analysis is restricted to these rare cases. Nonetheless, we would assume that a central pacemaker in the brain stem is also prevalent in individuals with a normal RSA and 1:2 or 1:3 coupling (two or three breaths during one RRI cycle). First results from the calculation of phase coupling (PLV) between BOLD oscillations from brain stem and RRI oscillations in the 0.1- to 0.15-Hz band support this assumption. With this method, it is possible to measure pacemaker activity in the brain stem independent of the breathing rate. This work is in progress.

It has to be noted that the level of state anxiety in the individuals with slow spontaneous breathing varied between AS = 14 and AS = 28 (possible range of AS scores: 10–40) and not every individual with elevated anxiety scores exhibited this kind of slow breathing. We therefore conclude that no clear relationship exists between slow spontaneous breathing and anxiety processing.

## Conclusion

1. Resting state BOLD oscillations from the cerebellum/brain stem can have alternating frequencies between 0.1 and 0.15 Hz, similar to those reported in RRI and respiration signals (Rassler et al., 2018).
2. Some BOLD signals from the cerebellum/brain stem can be influenced by vasomotion (basilar artery), while others may be associated with a central pacemaker activity in the brain stem. Consequently, the actual time of breathing onset as well as its neural source could be detected in BOLD signals.
3. Both BOLD signals with different origins recorded simultaneously characterize a common source, which supports the work of Perlitz et al. (2004).
4. The results suggest that there is evidence for an unconscious emotional breathing at 6–9/min (0.1–0.15 Hz), which – in a similar way as conscious resonance breathing at 6/min (0.1 Hz) promoting psychological well-being (Mather and Thayer, 2018) – also elevates HRV and may facilitate the processing of negative emotions.

## Data Availability

The datasets generated for this study are available on request to the corresponding author.

## Ethics Statement

This study was carried out in accordance with the recommendations of the 1964 Declaration of Helsinki with written informed consent from all subjects. All subjects gave written informed consent in accordance with the Declaration of Helsinki. The protocol was approved by the Ethics Committee at the University of Graz.

## Author Contributions

GP contributed to conceptualization and original draft. BR and AA helped in methodology, data processing, statistics, writing, and visualization. AS, BR, GS, WK, and JT reviewed and edited the manuscript.

### Conflict of Interest Statement

The authors declare that the research was conducted in the absence of any commercial or financial relationships that could be construed as a potential conflict of interest.

## Abbreviations

BOLD: Blood-oxygenation-level-dependent
ECG: Electrocardiogram
(f)MRI: (functional)Magnetic resonance imaging
HR: Heart rate
HRV: Heart rate variability
PFC: Prefrontal cortex
ROI: Region of interest
RRI: Beat-to-beat interval
RSA: Respiratory sinus arrhythmia.
